# ^31^P-MRS saturation transfer for assessing human hepatic ATP synthesis at clinical field strength

**DOI:** 10.1186/s41747-025-00588-9

**Published:** 2025-05-13

**Authors:** Marc Jonuscheit, Benedict Korzekwa, Michael Schär, Julian Mevenkamp, Stefan Wierichs, Pavel Bobrov, Theresia Sarabhai, Sabine Kahl, Michael Roden, Vera B. Schrauwen-Hinderling

**Affiliations:** 1 Institute for Clinical Diabetology, German Diabetes Center, Leibniz Center for Diabetes Research at Heinrich Heine University Düsseldorf, Düsseldorf, Germany; 2 German Center for Diabetes Research (DZD e.V.), Partner Düsseldorf, München-Neuherberg, Germany; 3 Division of Magnetic Resonance Research, Department of Radiology, Johns Hopkins University School of Medicine, Baltimore, United States of America; 4 Department of Radiology and Nuclear Medicine, Maastricht University Medical Center, Maastricht, The Netherlands; 5 Institute for Biometrics and Epidemiology, German Diabetes Center, Leibniz Center for Diabetes Research at Heinrich Heine University Düsseldorf, Düsseldorf, Germany; 6 Department of Endocrinology and Diabetology, Medical Faculty and University Hospital Düsseldorf, Heinrich Heine University Düsseldorf, Düsseldorf, Germany

**Keywords:** Adenosine triphosphate, Diabetes mellitus (type 1), Energy metabolism, Liver, Magnetic resonance spectroscopy

## Abstract

**Background:**

^31^P-magnetic resonance spectroscopy (MRS) saturation transfer (ST) allows for noninvasive investigation of liver energy metabolism by assessing flux rates of adenosine triphosphate (ATP) synthesis. However, this technique has rarely been applied at clinical field strengths because of long examination times and contamination from muscle tissue. Our aim was to establish a new method to robustly assess ATP synthesis using a clinical scanner.

**Methods:**

A prospective single-center study was performed (January 2023–August 2024) within the German Diabetes Study. We established a suitable ^31^P-MRS ST protocol, tested it *in vitro* and *in vivo* and assessed its reproducibility. We assessed the hepatic apparent spin-lattice relaxation time of inorganic phosphate (${T}_{1,{Pi}}^{{\prime} }$), equilibrium forward rate constant (${k}_{f}$), and forward ATP synthesis rate (${F}_{{ATP}}$) in nine control volunteers (CON) (six females) and eight patients (five females) with type 1 diabetes (T1D) and compared differences by ANOVA.

**Results:**

Reproducibility assessment in nine CON, aged 27 ± 4 years (mean ± standard deviation), yielded coefficients of variation for repeated measurements of 7.1% and 21.3% for ${T}_{1,{Pi}}^{{\prime} }$ and ${k}_{f}$, respectively. Group comparison revealed higher hepatic ${k}_{f}$ (0.34 ± 0.03 s^-1^ *versus* 0.16 ± 0.03 s^-1^; *p* = 0.001) and ${F}_{{ATP}}$ (35.3 ± 3.5 mM/min *versus* 16.4 ± 3.5 mM/min; *p* = 0.002) in CON than in T1D, aged 42 ± 15 years, respectively.

**Conclusion:**

This ^31^P-MRS ST method allowed for robust assessment of hepatic ATP synthesis at clinical field strength and was sensitive enough to detect differences between CON and T1D volunteers.

**Relevance statement:**

Noninvasive methods to investigate hepatic energy metabolism are urgently needed to evaluate liver health while preventing unnecessary biopsies. For broad clinical applicability, the robustness shown by the proposed method at clinical field strength is crucial.

**Trial registration:**

ClinicalTrials.gov: NCT01055093—Prospective study on diabetes mellitus and its complications in newly diagnosed adult patients (GDC), NCT01055093, Registered: 01/22/2010, https://clinicaltrials.gov/study/NCT01055093?term=NCT01055093&rank=1#study-overview.

**Key Points:**

- The proposed magnetic resonance spectroscopy method calculates hepatic ATP synthesis rates at clinical field strength.
- The protocol shows acceptable reproducibility and spectra without contamination from muscle.
- The method can detect differences between participants with type 1 diabetes and controls.

**Graphical Abstract:**

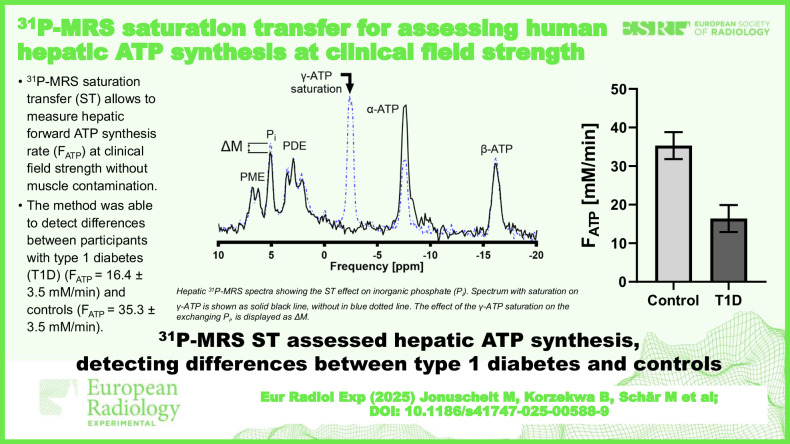

## Background

Intact mitochondrial function is required for sufficient adenosine triphosphate (ATP) production for cellular demands [1]. In the liver, oxidative capacity changes during the development of metabolic dysfunction-associated steatotic liver disease (MASLD) and steatohepatitis (MASH). In surgical biopsies, *ex vivo* measured energy metabolism is upregulated in steatotic livers, but is impaired in MASH and fibrosis, associated with excessive lipid accumulation and oxidative stress, likely contributing to worsening liver damage and disease progression [1, 2]. As percutaneous liver biopsies are only taken upon clinical indication, noninvasive techniques are pivotal for further studies. Moreover, given the recent U.S. Food and Drug Administration approval of the liver-selective thyroid hormone receptor ß-agonist, resmetirom, for MASH [3], mechanistic studies monitoring mitochondrial function in the liver and thereby treatment efficacy are important. While diagnosis and treatment of MASH in obesity and type 2 diabetes (T2D) is a well-documented unmet clinical need and people with MASH and T2D feature lower hepatic oxidative capacity [4], it is less well known that also type 1 diabetes (T1D) shows derangements of hepatic energy metabolism, that deserve further study.

^31^P-magnetic resonance spectroscopy (MRS) has been established for the detection of metabolite ratios and absolute concentrations [5, 6]. The few studies available show an impaired hepatic energy metabolism in people with T1D/T2D, indicated by decreased absolute concentrations of ATP and inorganic phosphate (P_i_) [7, 8]. Furthermore, the saturation transfer (ST) technique allows to observe the reaction kinetics of ATP synthesis [9], which has been previously applied in brain [10, 11], skeletal muscle [12, 13], and liver [14–16]. Up to now, the method was mainly applied at ultrahigh field (UHF) [16–21] and not at clinical field strength, because for the liver, spatial localization is mandatory, resulting in low signal-to-noise ratio (SNR) and long measurement times of > 2 h at clinical field strength. So far, the reproducibility of this method has only been assessed at UHF, and its variance during repeated measurements at clinical field strength is currently unknown.

The current study introduces “four repetition time ST” (FRiST) to robustly assess ATP synthesis using a clinical scanner with a high degree of spatial localization within 90 min. This method is based on TRiST (“triple repetition time ST”) introduced by Schär et al for measurements of creatine kinase reaction rates in human calf and heart [22]. After extensive phantom testing, the reproducibility of the method was evaluated and applied in participants with T1D to detect differences as compared to control volunteers.

## Methods

### Study design, volunteers and MRS system

This prospective, cross-sectional study was performed as a single-center study between January 2023 and August 2024, utilizing a subcohort from the ongoing German Diabetes Study (GDS) [23] (ClinicalTrials.gov: NCT01055093). The study was approved by the local ethics committee (Medical Faculty, Heinrich Heine University, Düsseldorf; ref#4508), and all participants provided written informed consent. Nine control volunteers were included for protocol optimization and determination of reproducibility, and nine T1D volunteers for clinical relevance assessment (Table 1). Inclusion and exclusion criteria are described in Supplementary Material S1. Participants fasted overnight and refrained from caffeine, alcohol, and strenuous exercise on the day preceding measurements. All examinations were performed on a clinical 3-T system (Philips Achieva dStream) using a quadrature surface coil [24].

**Table 1:** Participants’ characteristics

Volunteer group	Control volunteers	Type 1 diabetes	*p*-value
Number	9	8	
Sex (m/f)	3/6	3/5	1.000^a^
Age (years)	25.0 ± 3.43	42.1 ± 15.2	0.015*^b^
Weight (kg)	69.4 ± 11.5	74.5 ± 15.7	0.457^b^
Height (m)	1.70 ± 0.08	1.75 ± 0.11	0.395^b^
BMI (kg/m^2^)	23.8 ± 2.16	24.2 ± 2.65	0.734^b^
Data used for	Assessment of ${T}_{1,{Pi}}^{{\prime} }$, ΔM, ${k}_{f}$ and ${F}_{{ATP}}$ and reproducibility	Assessment of ${T}_{1,{Pi}}^{{\prime} }$, ΔM, ${k}_{f}$ and ${F}_{{ATP}}$	

All values are reported as numbers of participants or mean ± standard deviation

*ΔM* Difference of P_i_ between saturating and control irradiation, ${F}_{{ATP}}$ Forward adenosine triphosphate synthesis rate, ${k}_{f}$ Forward rate constant, ${T}_{1,{Pi}}^{{\prime} }$ Apparent spin-lattice relaxation time of P_i_

* *p* < 0.05 indicates statistical significance

^a^ Fisher’s test

^b^ Welch *t*-test

### ^31^P-MRS ST FRiST protocol

A detailed description of the *in vitro* and *in vivo* protocols is given in Supplementary Material S2. First, *in vitro* experiments were performed to optimize γ-ATP resonance saturation in the liver using a pulse train of delays alternating with nutations for tailored excitation (DANTE) [25] pulses. Two effective pulse schemes were identified with either 5 suppression bands, which were 9 Hz apart (M5D9) or 3 with 12 Hz (M3D12). The necessary DANTE subpulse flip angle β was determined to achieve < 5% γ-ATP residual. For *in vivo* validation of the protocols, the measurements were repeated in two male control volunteers aged 24 and 29 years, with body mass index (BMI) of 26.3 and 24.8 kg/m^2^.

In order to accelerate the determination of the apparent spin-lattice relaxation time of P_i_ (${T}_{1,{Pi}}^{{\prime} }$), a saturation recovery experiment with two-dimensional-localization and a range of five different repetition times (TRs) was tested in two control volunteers and checked which 3TRs best reflect the 5TR value.

All measurements were performed by two experienced spectroscopists (M.J. and B.K., with 6 and 4 years of experience, respectively), including segmentation of the liver and calculation of resonance frequency of the saturation pulse. Scout images were acquired and shimming was performed, using a dedicated shim tool, as described by Schär et al [26]. With this method, a ${B}_{0}$ map was acquired, the liver was manually segmented in 16 slices, and the resulting calculated ${B}_{0}$ shim was maintained for all measurements in that session. Mean SD linewidths of the water peak before and after shimming amounted to 47 Hz and 18 Hz, respectively, and the water peak was visually inspected to confirm a single, narrow, symmetrical peak. Transverse T_2_-weighted images acquired with multislice two-dimensional single-shot turbo spin-echo (TR/echo time 571/80 ms; 23 slices of 6-mm thickness, 1 mm gap, field of view 450 × 302 mm^2^) served together with coronal images from the scout images for further planning of the ^31^P-MRS measurements (Supplementary Fig. S1). To correctly apply the saturation pulse, the exact resonance frequencies of γ-ATP and P_i_ were determined from a nonlocalized ^31^P-MRS scan (Supplementary Material S3).

All ST experiments were acquired using a two-dimensional-localized “image-selected *in vivo* spectroscopy” [27] sequence with a 40 × 90 mm^2^ volume of interest (open in feet-head direction) and a hyperbolic secant adiabatic pulse for excitation and inversion (Supplementary Material S3). Four spectra were acquired with varying TRs and number of signal averages (${{TR}}_{{short}}\,$ = 0.7 s, ${{TR}}_{{center}}\,$ = 1.7 s, ${{TR}}_{{mirrored}}$ = 1.7 s, and ${{TR}}_{{long}}$ = 2.7 s). Further details of the acquisition procedure are listed in Supplementary Table S1, and a representative raw MRS spectrum acquired with TR 1.7 s is shown in Supplementary Fig. S2 [28].

In the T1D participants, three-dimensional-localized “image-selected *in vivo* spectroscopy” spectra were collected for quantification of absolute P_i_ concentration with correction for hepatic lipid content, as assessed by ^1^H-MRS as previously described [24].

### Data processing and quantification procedure

In line with best practices, all spectra were reviewed and processed by experienced spectroscopists (M.J., B.K., and S.W. with 6, 4, and 6 years of experience). To ensure good quality data, all spectra were analyzed in terms of SNR using the consensus definition ${SNR}=\frac{{Signal}}{{\sigma }_{{Noise}}}$ [28, 29]. After application of a 15 Hz Gaussian filter, the signal was defined as the fitted amplitude of the metabolite of interest and noise (σ) as standard deviation of the spectrum between 10 and 20 ppm (Supplementary Fig. S2) both in the frequency domain using a custom written MATLAB script (MathWorks Inc. R2021a, Natick, MA, USA). As criteria for good quality, spectra with SNR of γ-ATP < 4, P_i_ < 2.5, and minimal phosphocreatine (PCr) contamination (PCr to γ-ATP peak ratio > 0.15) were excluded. A sophisticated, fully automated, custom-written MATLAB script was used to analyze all ST spectra (Supplementary Material S4).

In the ^31^P-MRS ST experiments, the pseudo-first-order equilibrium forward exchange rate constant (${k}_{f}$) was calculated according to (Supplementary Material S5):

$${k}_{f}=\frac{1}{{T}_{1,{Pi}}^{{\prime} }}\left(1-\frac{{M}_{0,{Pi}}^{{\prime} }}{{M}_{0,{Pi}}}\right)$$

${T}_{1,{Pi}}^{{\prime} }$ was fitted from the signal amplitudes of the 3TR experiment using a mono-exponential equation. For assessment of the equilibrium (${M}_{0,{Pi}}$) and apparent longitudinal magnetization of P_i_ (${M}_{0,{Pi}}^{{\prime} }$), the corresponding P_i_ amplitude at ${{TR}}_{{mirrored}}$ and ${{TR}}_{{center}}$ were individually ${T}_{1}$ corrected for partial saturation by using ${T}_{1,{Pi}}$ of 730 ms obtained from literature [15] and the mean measured ${T}_{1,{Pi}}^{{\prime} }$ of the corresponding group. ${F}_{{ATP}}$ was calculated by multiplying ${k}_{f}$ with the corresponding P_i_ concentration. ^31^P-spectra for absolute P_i_ quantification were evaluated as previously described [24, 30]. Further details for the quantification process are given in Supplementary Material S4.

### Statistical analysis

All results are reported as individual values, mean ± standard error of the mean or mean [95% confidence interval], with the exception of age, weight, height, and BMI, reported as mean ± standard deviation. For the reproducibility study in control volunteers, the coefficient of variation (CV) was determined for repeated measurements of ${T}_{1,{Pi}}^{{\prime} }$, ΔM (difference in P_i_ between saturated and mirrored experiment), and ${k}_{f}$. Testing for outliers was performed using Grubbs’s test in R (version 4.0.5). For inter-group comparison ${T}_{1,{Pi}}^{{\prime} }$, ΔM, ${k}_{f}$, and ${F}_{{ATP}}$ were compared between the control and T1D participants, using ANOVA for generalized linear model without and with adjusting for covariates at a statistical significance level of *p* < 0.05. Sample size calculation used Cohen’s *d* method based on previously published data [21] and resulted in a power of 0.85 when investigating *n* = 8 participants per group, calculated using the POWER procedure in SAS software (SAS Institute Inc., Cary, NC, USA, Version 9.4) with α = 0.05, Cohen’s *d* = 1.6 and nominal power = 0.8. For deciding on the best TRs to determine ${T}_{1,{Pi}}^{{\prime} }$, the coefficient of determination (R^2^) was calculated using least squares regression without weighting and special handling for outliers.

## Results

### Participant characteristics and quality of spectra

**Fig. 1:**
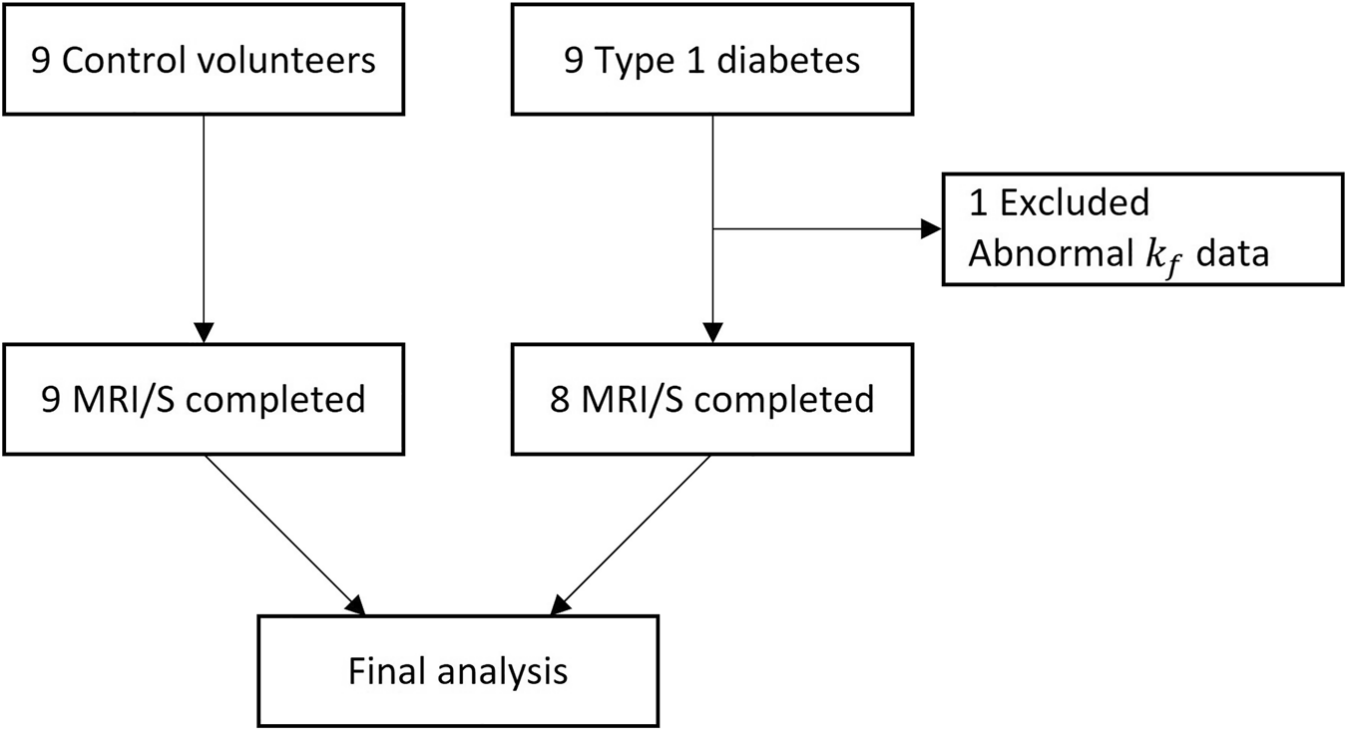
Flowchart of the study participants. ${k}_{f}$, Forward rate constant; MRS, Magnetic resonance spectroscopy

The characteristics were similar in both groups, except for higher mean age in the group of T1D (Table 1). The mean age of T1D patients was 42 ± 15 years (mean ± standard deviation), that of controls 25 ± 3 years (*p* = 0.015), BMI was 24 ± 3 kg/m^2^ *versus* 24 ± 2 kg/m^2^, respectively (*p* = 0.734). Three of eight T1D patients were males (38%), three of nine controls were males (33%). Valid MRS results were obtained from all participants, except for two people. One T1D patient was excluded from the analyses due to a physiologically impossible negative ${k}_{f}$ value, confirmed being an outlier by Grubbs’s test (Fig. 1). In a second T1D patient, a spectrum for absolute P_i_ concentration was excluded due to low SNR, but group average P_i_ was used to calculate ${F}_{{ATP}}$.

^31^P-MRS ST spectra showed mean SNRs of 5.7 ± 0.2, 5.8 ± 0.3, and 6.0 ± 0.2 for P_i_ in the saturated spectra using TR 0.7, 1.7, and 2.7 s, respectively. In the mirrored spectra, mean SNRs of 12.0 ± 0.4 and 6.2 ± 0.3 for γ-ATP and P_i_ were achieved. Absolute P_i_ quantification spectra had SNRs of 8.0 ± 0.8 and 6.3 ± 0.3 for γ-ATP and P_i_, respectively. PCr was completely absent or negligible with highest PCr/γ-ATP ratio of 11%, indicating good liver localization. ATP was fully saturated with residual γ-ATP < 5%, except for two spectra with 6% and 11% of residual γ-ATP. As almost the complete data set (26/28) had very good saturation (> 95%), no correction for partial saturation was applied. The suppression sideband in the mirrored spectrum (Supplementary Fig. S3) leads to a partial saturation of α-ATP, which, however, was considered as negligible based on the result of a previous study [31].

### Optimization of the FRiST method

**Fig. 2:**
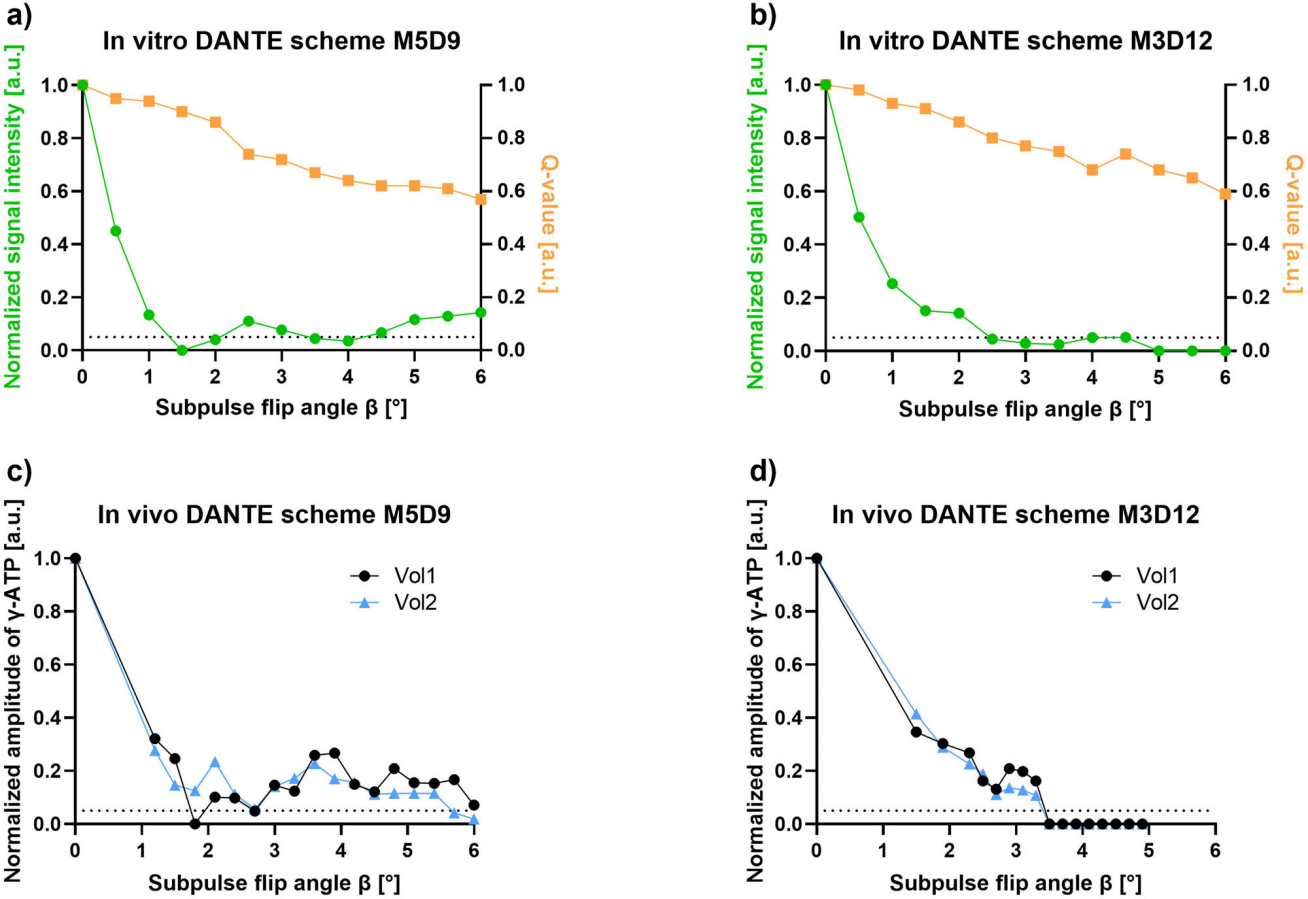
Amplitude modulation of the delays alternating with nutations for tailored excitation (DANTE) saturation pulse scheme. **a**, **b** *In vitro* results of the calculated normalized magnetization of γ-adenosine triphosphate (γ-ATP) with varying subpulse flip angle (β) at repetition time (TR) of 0.7 s for the scheme: (i) M5D9 (5 saturation bands each 9 Hz apart); and (ii) M3D12 (3 saturation bands each 12 Hz apart). **c**, **d** *In vivo* results of the same DANTE pulse train schemes for two control volunteers. The dashed line symbolizes a γ-ATP saturation of 5%. Q, Spillover; Vol, Volunteer

The *in vitro* testing for suitable DANTE pulse train schemes with the voxel of interest at a distance of 10 cm between coil and voxel of interest to simulate measurements in the liver are shown in Fig. 2a, b. In both tested pulse schemes, it was possible to saturate the γ-ATP moiety to < 5% of its original signal. For M5D9, two local minima areas were found (β = 1.5–2.0° and β = 3.5–4.0°), while the scheme M3D12 also showed two, but wider minima (β = 2.5–4.0° and β = 5.0–6.0°). The spillover (Q), representing the amount of signal decrease resulting from off resonance saturation, is constantly decreasing with higher flip angles, with a minimum of 59% of the initial signal amplitude at β = 6° for both schemes (see Fig. 2a, b). Application of these experiments in two control volunteers showed local minima at β = 1.8°, 2.7°, 5.7°, and 6.0° in one volunteer each for M5D9, while for M3D12 both volunteers exhibit a complete γ-ATP saturation for β > 3.5° (Fig. 2c, d) which is why M3D12 was further used in this study. Additionally, in order to keep Q high while maintaining complete saturation and minimizing specific absorption rate levels, the flip angle β = 4.0° was chosen. Using this protocol, Q amounted to 0.67 at β = 4.0° in both volunteers. A complete overview of the resulting DANTE saturation bands, including their sidebands in a typical (mirrored) ^31^P-MRS ST spectrum, is given in Supplementary Fig. S3. In the γ-ATP saturation experiments, the main saturation band is applied at -2.48 ppm with its sidebands far off from other ^31^P-resonances. In the mirrored experiment, the main saturation band is applied mirrored to the P_i_ resonance at 12.7 ppm.

**Fig. 3:**
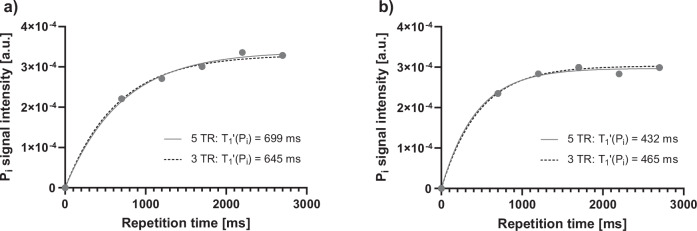
**a**, **b** Results of the saturation recovery experiment for determination of the apparent spin-lattice relaxation time of P_i_ (${T}_{1,{Pi}}^{{\prime} }$) during active saturation of γ-adenosine triphosphate (γ-ATP) for two control individuals. ${T}_{1,{Pi}}^{{\prime} }$ was determined by fitting five data points (dotted line) and by the 3TR method using only data points at 0.7, 1.7, and 2.7 s (solid line). P_i_, Inorganic phosphate; TR, Repetition time

**Fig. 4:**
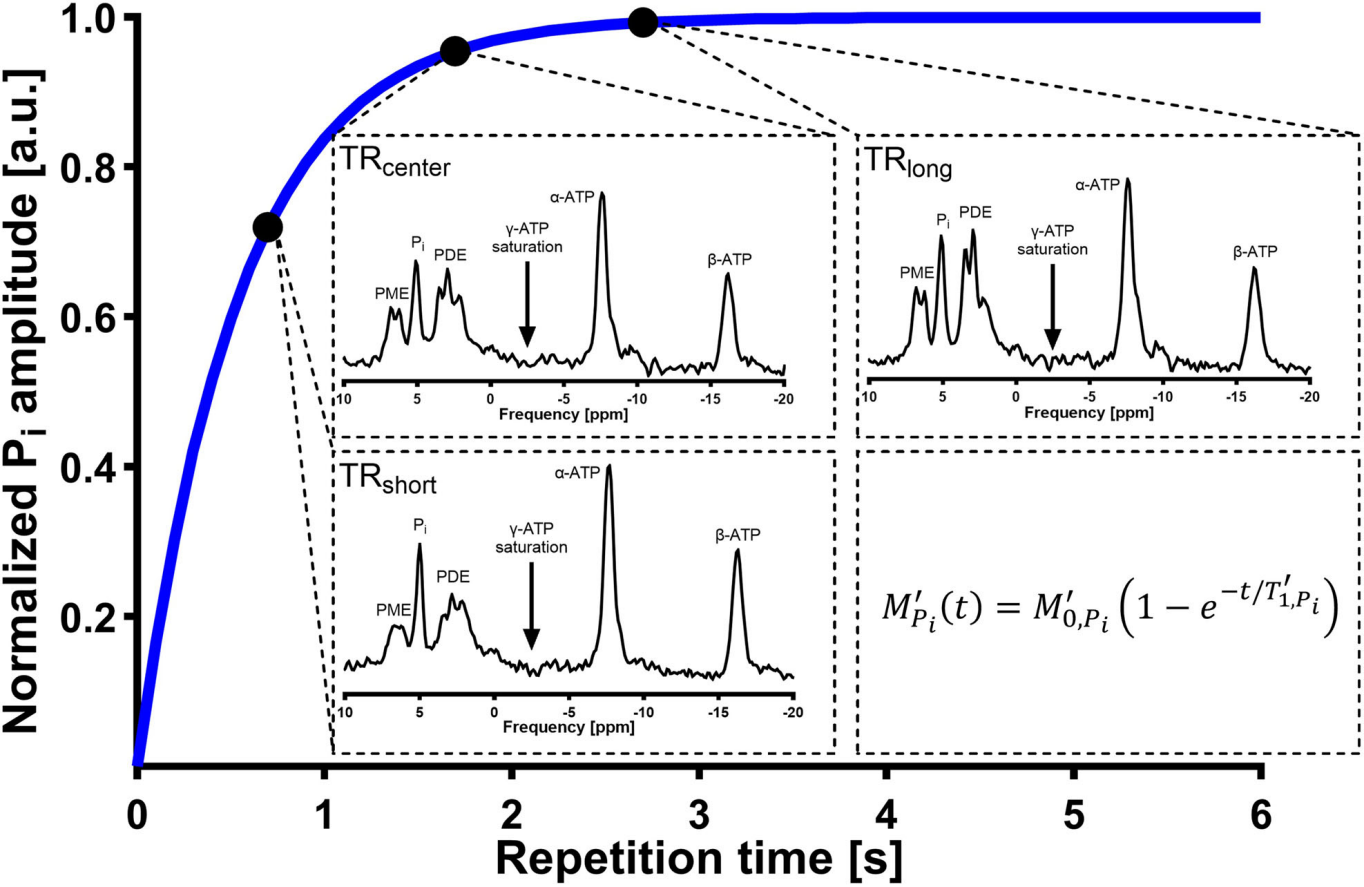
Determination of the apparent spin-lattice relaxation time of P_i_ (${T}_{1,{Pi}}^{{\prime} }$) by a saturation recovery experiment in a control volunteer. Active saturation on the γ-adenosine triphosphate (γ-ATP) resonance was applied at three different TRs (0.7, 1.7, and 2.7 s) and ${T}_{1,{Pi}}^{{\prime} }$ was obtained by fitting the inorganic phosphate (P_i_) amplitudes according to the displayed formula. The blue curve represents the relaxation behavior for ${T}_{1,{Pi}}^{{\prime} }$ = 520 ms. Spectra are apodized by a 10 Hz Gaussian filter. ATP, Adenosine triphosphate; PDE, Phosphodiesters; PME, Phosphomonoesters; TR, Repetition time

For shorter measurement time during ${T}_{1,{Pi}}^{{\prime} }$ determination, a set of 3TRs with the least deviation from a 5TR saturation recovery experiment was extracted for two control volunteers. The 5TR fit yielded a ${T}_{1,{Pi}}^{{\prime} }$ of 699 and 432 ms, respectively (solid lines in Fig. 3). Starting from five TRs, mono-exponential fits were executed with all TR combinations in order to find a TR triplet which is able to replicate the 5TR ${T}_{1,{Pi}}^{{\prime} }$. Here, fitting the combination of TR 0.7, 1.7, and 2.7 s yielded ${T}_{1,{Pi}}^{{\prime} }$ of 645 and 465 ms with R^2^ of 0.9993 and 0.9995, which differ by 8 and 9% from the five-point fit, respectively (dotted lines in Fig. 3), resulting in applying these TRs for all future measurements. A list of all TR sets for ${T}_{1,{Pi}}^{{\prime} }$ fitting including R^2^ can be reviewed in Supplementary Table S2. An example fit of the final TR set (TR 0.7, 1.7, and 2.7 s) and their position on the predicted ${T}_{1,{Pi}}^{{\prime} }$ relaxation curve is depicted in Fig. 4.

### Reproducibility of the method

The reproducibility of the method was assessed by performing a second measurement after full repositioning and resulted in mean CVs of 7.1%, 20.9%, and 21.3% for ${T}_{1,{Pi}}^{{\prime} }$, ΔM, and ${k}_{f}$, respectively, and decreases to 14.0% and 14.6% for ΔM and ${k}_{f}$ when omitting one datapoint which was identified as statistical outlier (*p* = 0.006, control volunteer #7). Table 2 lists all individual data for the reproducibility measurements.

**Table 2:** Individual data from the control volunteer group saturation transfer experiments

Volunteer	${\boldsymbol{T}}_{{\bf{1}},{\boldsymbol{Pi}}}^{{\prime}}$ (ms)	${\boldsymbol{T}}_{{\bf{1}},{\boldsymbol{Pi}}}^{{\prime}}$ (ms)	${\boldsymbol{T}}_{{\bf{1}},{\boldsymbol{Pi}}}^{{\prime}}$ (ms) (% CV)	ΔM (%)	ΔM (%)	ΔM (%) (% CV)	${\boldsymbol{k}}_{\boldsymbol{f}}$ (s^-1^)	${\boldsymbol{k}}_{\boldsymbol{f}}$ (s^-1^)	${\boldsymbol{k}}_{\boldsymbol{f}}$ (s^-1^) (% CV)
1	500	467	484 ± 17 (4.8%)	13.6	14.3	14.0 ± 0.4 (4.0%)	0.27	0.31	0.29 ± 0.02 (8.8%)
2	591	550	571 ± 21 (5.1%)	14.3	15.8	15.1 ± 0.8 (7.1%)	0.24	0.29	0.26 ± 0.02 (12.1%)
3	469	427	448 ± 21 (6.6%)	17.5	13.9	15.7 ± 1.8 (16.4%)	0.37	0.33	0.35 ± 0.02 (9.8%)
4	540	443	492 ± 49 (14.0%)	14.9	15.9	15.4 ± 0.5 (4.7%)	0.28	0.36	0.32 ± 0.04 (18.5%)
5	618	519	569 ± 50 (12.3%)	17.5	14.9	16.2 ± 1.3 (11.3%)	0.28	0.29	0.29 ± 0.001 (1.0%)
6	385	423	404 ± 19 (6.7%)	19.1	16.9	18.0 ± 1.1 (8.5%)	0.50	0.40	0.45 ± 0.05 (15.1%)
7	388	389	389 ± 1.0 (0.2%)	6.7	21.9	14.3 ± 7.6 (75.5%)	0.17	0.56	0.37 ± 0.20 (75.3%)
8	566	548	557 ± 9.0 (2.3%)	13.3	18.3	15.8 ± 2.5 (22.1%)	0.24	0.33	0.28 ± 0.05 (24.3%)
9	482	573	528 ± 46 (12.2%)	6.5	11.4	9.0 ± 2.4 (38.4%)	0.14	0.20	0.17 ± 0.03 (26.8%)
Mean	504	482	493 ± 26 (7.1%)	13.7	15.9	14.8 ± 2.0 (20.9%)	0.28	0.34	0.31 ± 0.05 (21.3%)
Mean*	519	494	506 ± 29 (8.0%)	14.6	15.2	14.9 ± 1.3 (14.0%)	0.29	0.31	0.30 ± 0.03 (14.6%)

Values are reported as mean or mean ± standard error of the mean and coefficient of variation (CV) in parentheses

Mean* mean without the outlier volunteer #7, *ΔM* Percentage difference in P_i_ amplitude between saturating and control irradiation, ${k}_{f}$ Forward rate constant, ${T}_{1,{Pi}}^{{\prime}}$ Apparent spin-lattice relaxation time of P_i_

### Application of FRiST in individuals with T1D and comparison to control volunteers

**Fig. 5:**
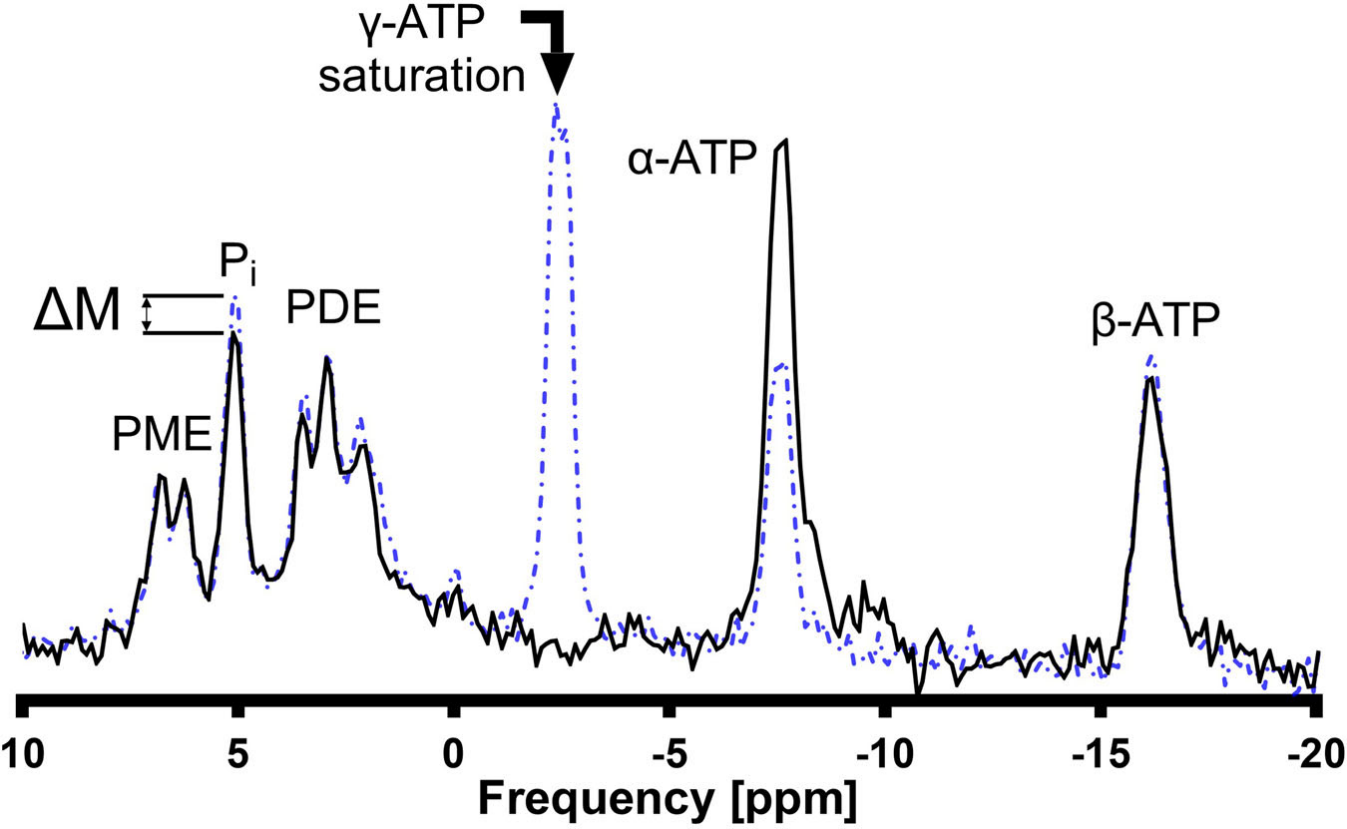
Hepatic ^31^P-magnetic resonance spectroscopy saturation transfer (ST) spectra showing the ST effect on inorganic phosphate (P_i_). The black arrow indicates the frequency where the delays alternating with nutations for tailored excitation saturation pulse scheme was applied (directly on γ-ATP), and the resulting spectrum is shown as a solid black line. The spectrum of a second experiment performed with the same saturation pulse scheme applied at the mirrored frequency is shown in blue dotted line. The effect of the γ-ATP saturation on the exchanging P_i_, is displayed as ΔM. Spectra are apodized by a 5 Hz Gaussian filter. ΔM percentage difference in P_i_ amplitude between saturating and control irradiation. ATP, Adenosine triphosphate; PDE, Phosphodiesters; PME, Phosphomonoesters

Representative spectra of the hepatic ST experiment at TR 1.7 s with saturation on γ-ATP (dashed blue line) and mirrored frequency (solid black line) are shown in Fig. 5. As with the ${T}_{1,{Pi}}^{{\prime} }$ measurements (Fig. 4), the γ-ATP resonance is completely suppressed in the saturation experiment and residual PCr signal is absent indicating a high degree of signal localization.

**Fig. 6:**
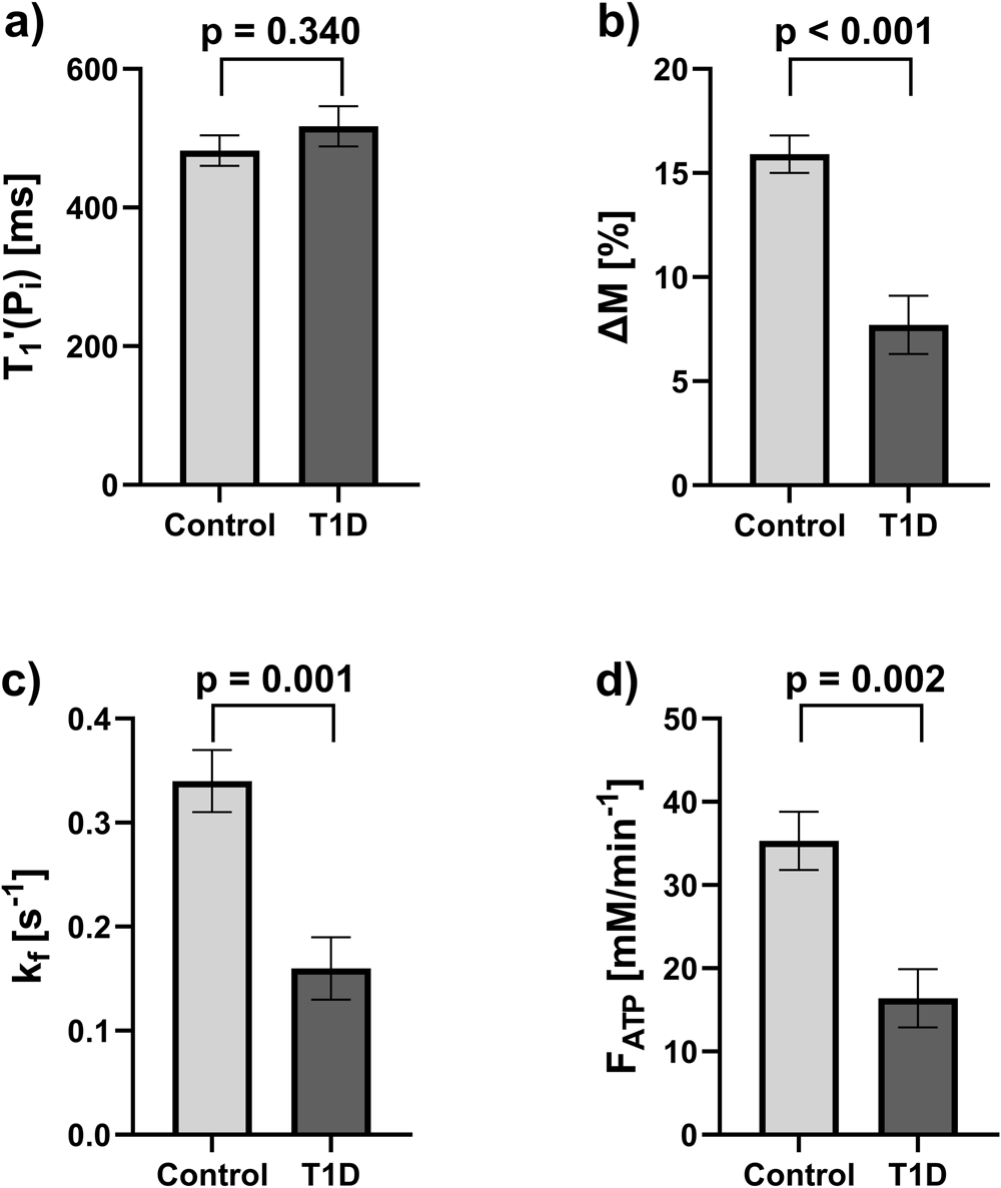
Results of the comparison of subgroups of control volunteers and type 1 diabetes (T1D) hepatic saturation transfer experiments. Comparison is shown for: (**a**) the apparent spin-lattice relaxation time of P_i_ (${T}_{1,{Pi}}^{{\prime} }$); (**b**) the percentage difference in P_i_ amplitude between saturating and control irradiation (ΔM); (**c**) the forward rate constant (${k}_{f}$); and (**d**) the forward ATP synthesis rate (${F}_{{ATP}}$). All graphs show mean ± standard error of the mean

We found no evidence of a difference in ${T}_{1,{Pi}}^{{\prime} }$ between the participants with (517 ± 29 ms) and without T1D (482 ± 22 ms, *p* = 0.340) (Fig. 6). Individuals with T1D had markedly lower ΔM with 7.7 ± 1.4% *versus* control volunteers with 15.9 ± 0.9% (*p* < 0.001) and in line, ${k}_{f}$ was also lower with 0.16 ± 0.03 s^-1^ *versus* 0.34 ± 0.03 s^-1^ (*p* = 0.001), respectively (Fig. 6). For the calculation of the forward ATP synthesis rate, P_i_ concentration is required. In the T1D group, the individual absolute concentrations of P_i_ were determined by using the phantom-replacement technique, which yielded P_i_ = 1.70 ± 0.11 mM and a hepatic lipid content of 0.67 ± 0.07%, resulting in ${F}_{{ATP}}$ of 16.4 ± 3.5 mM/min. For controls, a fixed value for P_i_ of 1.73 mM was assumed as determined in a previous study [24], and subsequently, a mean ${F}_{{ATP}}$ of 35.3 ± 3.5 mM/min was obtained. Data is summarized for both groups in Table 3. Additional analysis with adjustment for age, sex, and BMI still showed the same significant differences (Supplementary Table S3).

**Table 3:** Determined ^31^P-magnetic resonance spectroscopy parameters for the subgroups of control volunteers and type 1 diabetes

	Control volunteers	Type 1 diabetes	*p*-value
Number	9	8	
${T}_{1,{Pi}}^{{\prime} }$ (ms)	482 [431; 533]	517 [449; 585]	0.340
∆M (%)	15.9 [13.7; 18.2]	7.7 [4.3; 11.0]	< 0.001*
${k}_{f}$ (s^-1^)	0.34 [0.26; 0.42]	0.16 [0.08; 0.23]	0.001*
${F}_{{ATP}}$ (mM/min)	35.3 [27.3; 43.3]	16.4 [8.2; 24.7]	0.002*

All values are reported as numbers of participants or mean [95% CI]

*ΔM* Percentage difference in P_i_ amplitude between saturating and control irradiation, ${F}_{{ATP}}$ Forward adenosine triphosphate synthesis rate, ${k}_{f}$ Forward rate constant, *P*_*i*_ Inorganic phosphate, ${T}_{1,{Pi}}^{{\prime} }$ Apparent spin-lattice relaxation time of P_i_

* *p* < 0.05 indicates statistical significance

## Discussion

This study developed FRiST, a robust ^31^P-MRS ST method, to investigate hepatic energy metabolism on a clinical 3-T system. FRiST was able to: (1) successfully quantify hepatic ${k}_{f}$ and ${F}_{{ATP}}$ with acceptable reproducibility; (2) provide high spectral quality and purely hepatic measurements; (3) obtain results which are comparable to data acquired at UHF; and (4) detect significant changes in hepatic ${k}_{f}$ and ${F}_{{ATP}}$ between patients with T1D and controls (the significance remained when adjusting for age, sex, and BMI). These results, comparable to those that can be obtained at UHF [20, 21], show that the FRiST method is sensitive enough to be applied in future clinical studies.

The protocol optimizes the acquisition scheme, shimming, and spatial localization to obtain high spectral quality while keeping the total acquisition time to 90 min, allowing to assess purely hepatic fluxes, which is shown by minimal PCr signal. To reduce the acquisition time, FRiST uses a 3TR saturation recovery method to obtain ${T}_{1,{Pi}}^{{\prime} }$ instead of a 10-point inversion recovery approach [14, 15] and allows dual use of the TR 1.7 s spectrum for both, ${T}_{1,{Pi}}^{{\prime} }$ and ΔM assessment. FRiST shows acceptable CVs in repeated measurements. To the best of our knowledge, no data on the reproducibility of ST protocols in the liver at 3-T has been published so far, despite some reports of applying ST to the liver at 3-T. However, these protocols were very long and required two separate sessions [15] or the signal was not confined to the liver [14].

ST applied at UHF showed increased sensitivity compared to 3-T, with greater signal intensity differences (ΔM) of 21% *versus* 15%. Valkovič [20] reported CVs of 11.2% and 9.0% for ${T}_{1,{Pi}}^{{\prime} }$ and ${k}_{f}$ at 7-T in six controls. The CVs presented here were only slightly higher, with one outlier significantly affecting the mean CV of ${k}_{f}$ (CV_No.7_ = 75.3%). Excluding this outlier, confirmed by Grubbs’s test, would reduce ${k}_{f}$’s CV to 14.6%. It is unclear what the source of error was. Biological factors seem unlikely, possibly breathing artifacts may have influenced the outcome. While UHF offers clear advantages in spectral quality and reduced measurement time, it is usually not available for wider application in a clinical setting; the possibility to perform these measurements at clinical field strength was crucial for us and the driver to optimize the protocol at 3-T.

At clinical field strengths, hepatic ${k}_{f}$ and ${F}_{{ATP}}$ have been previously reported in control volunteers [14, 15] and in T2D [32]. The results for ${T}_{1,{Pi}}^{{\prime} }$ and ${k}_{f}$ in control participants are in good agreement with literature, where a range from 520 ± 20 ms to 580 ± 60 ms for ${T}_{1,{Pi}}^{{\prime} }$ and 0.27 ± 0.03 s^-1^ to 0.3 ± 0.02 s^-1^ for ${k}_{f}$ was found [14, 15]. Also ${F}_{{ATP}}$ results fall within the reported range, but literature shows more variation from 29.5 ± 1.8 mM/min [15] to 48.6 ± 7.4 mM/min [14] due to differences in assumed P_i_ concentrations. The P_i_ concentration used here (1.73 mM) agrees with the values reported by Schmid et al (1.64 mM) [15], but is much lower than the values reported by Buehler et al [14] (3.0 mM), explaining differences in ${F}_{{ATP}}$ across studies.

At 7-T, ${k}_{f}$ was found to be lower in people with MASH [19, 20] and in one study on T1D [21]. For T1D, their reported mean ${k}_{f}$ value of 0.17 ± 0.03 s^-1^ [21] is in excellent agreement with the findings of the current study (${k}_{f}\,$ = 0.16 ± 0.03 s^-1^). The significantly reduced ${k}_{f}$ and ${F}_{{ATP}}$ in T1D may indicate an impaired mitochondrial function. It is worth mentioning that this reduction occurred despite the very low hepatic lipid content in T1D in our study (0.67 ± 0.07%), agreeing with findings of Wolf et al (2.1 ± 0.4%) [21]. This is in contrast to results in T2D and MASLD/MASH, where increased hepatic lipid content often correlates with reduced ${F}_{{ATP}}$ [19, 20]. Of note, the decrease in ${F}_{{ATP}}$ was primarily shown in people with MASH, but not in people with MASL alone [19, 20] and one should note that the phenotype in T1D is quite different from MASLD/MASH with a decrease in ${F}_{{ATP}}$ while hepatic lipid content and liver enzymes are expected to be in the normal range.

When applying FRiST at 3-T, we confirmed lowered ${k}_{f}$ in T1D, showing that the method is sensitive enough to noninvasively detect clinical differences between control volunteers and people with T1D.

Since the FRiST method was developed at clinical field strength, it is possible to be integrated into clinical workflows as its noninvasive approach represents a valuable alternative to invasive liver biopsies. Furthermore, monitoring of treatment responses in MASLD/MASH and also risk stratification in MASLD may arise as a future application of the method, as changes in ${F}_{{ATP}}$ may be an early indicator of progression of liver disease, as shown by the decrease in ${k}_{f}$ and ${F}_{{ATP}}$ between MASL and MASH [19]. Being a quantitative measurement of hepatic ${F}_{{ATP}}$, it may also contribute to a more personalized treatment therapy for people with hepatic disorders. However, it should be noted that the duration of FRiST amounts to 90 min, preventing the method from being routinely implemented as an additional measurement into standard MRI/S workflows. Future work may focus on speeding up the measurements without impairing data quality.

Although we successfully detected significant changes in ${F}_{{ATP}}$ between T1D and controls, this study has several limitations. First, the interpretation of ST results is complex, with studies demonstrating that assessed metabolic fluxes in human skeletal muscle overestimate oxidative ATP synthesis [33]. ATP is produced through both anaerobic glycolysis and aerobic oxidative phosphorylation, so that ${F}_{{ATP}}$ measurements will include a significant glycolytic component [33, 34]. Reactions near equilibrium have a larger impact, as forward and backward reactions occur simultaneously, determining the unidirectional flux rather than the net flux. While ST data does not directly measure purely oxidative net ATP synthesis, ${k}_{f}$ and ${F}_{{ATP}}$ remain sensitive markers of energy metabolism, mostly changing in parallel with other related metabolic measures [35]. Second, we used mean values of P_i_ concentration from a previous study to calculate ${F}_{{ATP}}$ in control participants, rather than measuring individual absolute P_i_ concentration. However, P_i_ concentration in control volunteers typically shows only little variation, and the used concentration comes from a group with similar age and BMI. Third, the relatively small sample size used (nine controls, eight T1D) may limit the generalizability of our findings. Larger cohort studies need to be performed in the future to investigate milder clinical phenotypes and validate these results in a broader population. Future research should aim to provide a more comprehensive metabolic profile of the volunteers with more detailed clinical characterization, including assessment of additional metabolic markers such as HbA1c, insulin resistance, and fibrosis markers, as well as consideration of medication.

In conclusion, we developed FRiST, a new noninvasive ^31^P-MRS ST method to assess hepatic ${F}_{{ATP}}$ on a clinical scanner. This method demonstrates acceptable reproducibility, which allows meaningful sample size calculations for future biomedical research and clinical studies. FRiST demonstrates a sensitivity allowing to detect a decrease in ${F}_{{ATP}}$ in people with T1D compared to control volunteers and shows potential as a noninvasive tool for enhancing the understanding of liver diseases and improving diagnosis and monitoring of treatment.

## Supplementary information

**Additional file 1: Supplementary Fig. S1:** Planning of the FRiST protocol. a) Coronal and b) transverse MRI slices of the liver of a 30 years old control volunteer showing coil placement and position of the 2D-voxel of interest (VOI) (open in feet-head direction). White dots within the coil housing show position of reference spheres. **Supplementary Fig. S2:** Representative raw hepatic phosphorus magnetic resonance spectroscopy spectrum. No postprocessing was performed except zero order phasing. The spectral region between 10 ppm and 20 ppm served to determine the noise for calculation of signal-to-noise for spectral quality assessment. **Supplementary Fig. S3:** Position of the delays alternating with nutations for tailored excitation (DANTE) saturation bands with m = 3 saturation bands which were δ = 12 Hz (M3D12) apart on an entire hepatic phosphorus magnetic resonance spectroscopy spectrum. In the saturation experiment, the saturation pulse was centered on the γ-ATP resonance at ~-2.48 ppm (saturation bands displayed in red). Two aliased saturation bands (~-23.6 ppm and ~18.7 ppm) occur every ~21.2 ppm (1/τ = 1100 Hz) from the center of saturation frequency due to a DANTE subpulse duration of τ = 0.91 ms. In the mirrored experiment, the mirrored saturation frequency was set to ~12.7 ppm resulting in aliased saturation bands at ~33.82 ppm (not shown) and ~-8.5 ppm (blue dashed lines). Note that in the mirrored experiment α-ATP is partly affected by the aliased sideband resulting in a decreased signal amplitude. For better representation, the spectrum is apodized with a 5 Hz Gaussian filter. **Supplementary Table S1:** MRSinMRS checklist [14]. **Supplementary Table S2:** 3TR *versus* 5TR. Results of apparent spin-lattice relaxation time (*T*′_1,*Pi*_) fitting of a saturation experiment to find the best suited three repetition time (TR) set. Two control volunteers underwent a saturation recovery experiment consisting of five TRs. The experimentally determined *T*′_1,*Pi*_ together with the calculated coefficient of determination (R^2^) were compared to find the most accurate set of three TRs. **Supplementary Table S3:** Calculated phosphorus-MR spectroscopy parameters for the subgroups of control volunteers and type 1 diabetes using ANOVA adjusted for age, sex and BMI.

## Data Availability

The datasets used and/or analyzed during the current study are available from the corresponding author on reasonable request.

## Abbreviations

${F}_{{ATP}}$: Forward ATP synthesis rate
${T}_{1,{Pi}}^{{\prime} }$: Apparent spin-lattice relaxation time of inorganic phosphate
${k}_{f}$: Equilibrium forward rate constant
ATP: Adenosine triphosphate
BMI: Body mass index
CV: Coefficient of variation
DANTE: Delays alternating with nutations for tailored excitation
FRiST: Four repetition time saturation transfer
MASH: Metabolic dysfunction-associated steatohepatitis
MASLD: Metabolic dysfunction-associated steatotic liver disease
MRS: Magnetic resonance spectroscopy
PCr: Phosphocreatine
P_i_: Inorganic phosphate
SNR: Signal-to-noise ratio
ST: Saturation transfer
T1D: Type 1 diabetes
T2D: Type 2 diabetes
TR: Repetition time
UHF: Ultrahigh field
