# Supplementary material for: 31P-MRS saturation transfer for assessing human hepatic ATP synthesis at clinical field strength
Source: Eur Radiol Exp. 2025 May 13;9:51. doi: 10.1186/s41747-025-00588-9 (PMC12075714; doi:10.1186/s41747-025-00588-9)
Supplement: Supplementary file 1 — Additional file 1: Supplementary Fig. S1: Planning of the FRiST protocol. a) Coronal and b) transverse MRI slices of the liver of a 30 years old control volunteer showing coil placement and position of the 2D-voxel of interest (VOI) (open in feet-head direction). White dots within the coil housing show position of reference spheres. Supplementary Fig. S2: Representative raw hepatic phosphorus magnetic resonance spectroscopy spectrum. No postprocessing was performed except zero order phasing. The spectral region between 10 ppm and 20 ppm served to determine the noise for calculation of signal-to-noise for spectral quality assessment. Supplementary Fig. S3: Position of the delays alternating with nutations for tailored excitation (DANTE) saturation bands with m = 3 saturation bands which were δ = 12 Hz (M3D12) apart on an entire hepatic phosphorus magnetic resonance spectroscopy spectrum. In the saturation experiment, the saturation pulse was centered on the γ-ATP resonance at ~-2.48 ppm (saturation bands displayed in red). Two aliased saturation bands (~-23.6 ppm and ~18.7 ppm) occur every ~21.2 ppm (1/τ = 1100 Hz) from the center of saturation frequency due to a DANTE subpulse duration of τ = 0.91 ms. In the mirrored experiment, the mirrored saturation frequency was set to ~12.7 ppm resulting in aliased saturation bands at ~33.82 ppm (not shown) and ~-8.5 ppm (blue dashed lines). Note that in the mirrored experiment α-ATP is partly affected by the aliased sideband resulting in a decreased signal amplitude. For better representation, the spectrum is apodized with a 5 Hz Gaussian filter. Supplementary Table S1: MRSinMRS checklist [14]. Supplementary Table S2: 3TR versus 5TR. Results of apparent spin-lattice relaxation time (T′1,Pi) fitting of a saturation experiment to find the best suited three repetition time (TR) set. Two control volunteers underwent a saturation recovery experiment consisting of five TRs. The experimentally determined T′1,Pi together with the calc [file 41747_2025_588_MOESM1_ESM.pdf]

^{31}P-MRS saturation transfer for assessing human hepatic ATP synthesis at clinical field strength

ELECTRONIC SUPPLEMENTARY MATERIAL

Eur Radiol Exp (2025) Jonuscheit M, Korzekwa B, Schär M, et al.

Supplemental material S1:

Inclusion and exclusion criteria

Cohort profile: The German Diabetes Study (GDS)

The GDS is an ongoing prospective observational study comprising intensive phenotyping within 12 months after clinical diagnosis, at 5-year intervals for at least 20 years and annual telephone interviews in between. The study is performed according to the Declaration of Helsinki, approved by the ethics committee of the University of Düsseldorf and was registered at Clinicaltrials.gov (Identifier number: NCT01055093). A detailed description can be found in Szendroedi et al [1].

<u>Inclusion criteria</u>

a) Age of 18–69 years
b) Type 1 diabetes mellitus
    a. Diagnosis of T1D, diabetes onset within the last 12 months.
    b. T1D diagnosis based on ketoacidosis or immediate insulin requirement, presence of at least one islet cell directed autoantibody or C- peptide levels below detection limit
c) Healthy volunteers
    a. No diagnosis of diabetes

<u>Exclusion criteria</u>

a) Diabetes due to specific causes ("Type 3"; e.g., pancreoprive diabetes, genetically-induced diabetes)
b) Gestational diabetes, pregnancy
c) Poor glycemic control (HbA1c >9.0 %)
d) Hyperlipidemia (triglycerides and low- density lipoproteins ≥double upper reference limit)
e) Heart failure (New York Heart Association class ≥II)
f) Renal disease (serum creatinine ≥1.6 mg/dL)
g) Clinically relevant Liver disease (aspartate aminotransferase and/or alanine aminotransferase and/or gamma glutamyltransferase ≥double upper reference limit)
h) Peripheral artery occlusive disease stage IV
i) Venous thromboembolic events
j) Anemia or blood donation
k) Participation in a clinical study within the past 3 months

l) Acute infection, increased leukocytes, immunosuppressive therapy, autoimmune diseases, infection with human immunodeficiency virus, other severe diseases (e.g., active cancer disease)

m) Psychiatric disorders

n) Specific exclusion criteria for MRI/S studies
    a. pacemaker
    b. metallic and magnetic implants
    c. claustrophobia

Supplemental material S2:

In vitro & in vivo MR protocols for establishing FRiST

Frequency selective saturation using DANTE pulses

In order to saturate the γ-ATP resonance over its full linewidth in the human liver, the amplitude of the delays alternating with nutations for tailored excitation (DANTE) pulse train is modulated according to the flip angle function $\alpha(k)$ to produce multiple adjacent suppression bands [2, 3]:

$$\alpha(k) = \beta \sum_{n=0}^{m-1} \cos\left(2\pi\left(n - \frac{m-1}{2}\right)k\tau\delta\right)$$

In the above formula k denotes the number of subpulses, m is the number of suppression bands, δ represents the frequency separation in Hz between the individual suppression bands, β denotes the average subpulse flip angle per suppression band and τ represents the repetition time of the subpulses.

Using such short hard pulses allows for a narrow suppression band at a specific frequency. By splitting the applied irradiation into subpulses aliased suppression bands occur in the spectrum at frequency intervals set as the inverse of τ. In order to avoid any potential influence on the peak of interest (P_i), τ = 0.91 ms was chosen to generate aliased suppression bands 1/τ = 1100 Hz apart from the γ-ATP resonance which is far from any ^{31}P-metabolite and especially P_i (frequency offset γ-ATP ↔ P_i = ~400 Hz at 3T).

To find an optimal saturation scheme for successful saturation of the γ-ATP resonance in the liver, first in vitro experiments were performed. For this, a cylindrical 3 liter bottle containing 50 mM dipotassium phosphate (K_2HPO_4) served as phantom and a 2D-ISIS [4] localization sequence was used to test different DANTE pulse train schemes at a depth of 10 cm between coil and center of the voxel of interest (VOI) using a repetition time (TR) of 0.7 s, number of signal averages (NSA) 256, t_{exp} ~3 min. Here, two saturation schemes were tested using m = 5 suppression bands which were δ = 9 Hz apart (M5D9) and m = 3 with δ = 12 Hz (M3D12), respectively. In both experiments the subpulse flip angle β was varied between 0° and 6° in +0.5° steps in order to determine the minimal necessary β to saturate γ-ATP to < 5% of its unsaturated peak intensity. Additionally, the same β flip angle series was repeated with saturation at the mirrored frequency and finally, one spectrum without any saturation was acquired in order to calculate the spillover Q, representing the amount of signal decrease resulting from the presence of the saturation scheme at the respective pulse power. Q was calculated as the ratio between M_0 of the resonance of interest (P_i) with and without saturation [5]. Based on the results of the in vitro experiments, a similar β-series was performed in vivo to confirm the low power β-values obtained in vitro and also confirm good saturation of γ-ATP

in vivo. Therefore, the scheme M5D9 was examined between β = 1.2°–6.0° in +0.3° steps and the second scheme M3D12 was evaluated between β = 1.5°–2.3° in +0.4° steps and 2.3°–4.9° in +0.2° steps. Two control volunteers male volunteers (age 26.5 ± 3.5 years, BMI 25.7 ± 0.9 kg/m^2) underwent all measurements. Due to the long duration of this experiment, all experiments were performed with the shortest TR (TR 0.7 s) and NSA were limited to 512, which resulted in a measurement time of ~6 min per spectrum.

After evaluating the results, the protocol with the DANTE pulse train scheme that yielded the best outcome was applied together with the optimal β-value in both volunteers in a separate session to determine the Q-values of the chosen parameters. Here, spectra were acquired at mirrored saturation frequency with and without active saturation at TR 6 s, NSA 128, t_{exp} ~12.8 min.

Optimization of $T'_{1,Pi}$ determination

In order to determine the apparent spin-lattice relaxation time of P_i ($T'_{1,Pi}$), a saturation recovery experiment with 2D-localization and a range of different TRs was tested. Initial experiments showed that TR = 700 ms was the shortest successful TR for the DANTE saturation schemes and the following five TRs were applied and tested in 2 volunteers: TR 700, 1200, 1700, 2200 and 2700 ms with 1368, 840, 564, 496, 472 and 464 NSA (t_{exp} ~68 min), respectively. NSAs for each TR were adapted in order to keep SNR stable. Subsequently, it was tested to what extent data quality of $T'_{1,Pi}$ determination is compromised when only three of the five TRs are measured and which of the three TRs can best be chosen for reliable $T'_{1,Pi}$ determination.

Choice of TR for detecting the saturation effect

For assessment of the equilibrium ($M_{0,Pi}$) and apparent longitudinal magnetization of P_i ($M'_{0,Pi}$), a TR of 1.7 s was chosen based on reported values for $T'_{1,Pi}$ of 520 ms [6] or 580 ms [7] as well as $T_{1,Pi}$ of 730 ms [6] thereby keeping a good compromise between signal strength and measurement time. Because the expected difference in P_i amplitude (ΔM) between experiments is small (about 15%) [6] 768 NSA were acquired for each spectrum to achieve excellent SNR.

Supplemental material S3:

Extended MRI/S protocol parameters for FRiST

B_0 shimming

A three-dimensional (3D) isotropic fast field echo (FFE) was acquired during a single breath hold to acquire a B_0 map (field of view (FOV) 350 × 292 × 160 mm^3, acquisition voxel size 5 × 5 × 10 mm^3, reconstruction voxel size 2.43 × 2.43 × 10 mm^3, 16 transverse slices, t_{exp} 12 s).

Nonlocalized spectroscopy and calculation of frequency offsets for the FRiST experiment

Nonlocalized spectroscopy was applied with a 3.83 ms hyperbolic secant (HS) adiabatic half passage pulse for excitation (excitation bandwidth 1.63 kHz) TR 10 s, NSA 4, total acquisition time ~1 min, number of sample points (N) 512, spectral bandwidth (BW) 3 kHz, excitation pulse center ~1.0 ppm).

The unlocalized spectrum was used for determination of the individually frequencies of γ-ATP and P_i, in order to i) set the transmitter frequency of the successive ST experiments exactly in between both resonances, ii) calculate the participant specific frequency offset of the saturation pulse for the on-resonance experiments and iii) determine the frequency offset for the saturation pulse in case of the mirrored experiment.

FRiST experiment

All ST experiments were acquired using a 2D-localized image-selected in vivo spectroscopy (ISIS) [4] sequence with a 40 × 90 mm^2 voxel of interest (VOI) and a 3.83 ms HS adiabatic pulse for excitation (excitation bandwidth 1.63 kHz), N 512 and BW 3 kHz. The duration of the HS inversion pulse for slice selection was 3.9 ms, HS inversion bandwidth 3.18 kHz. The TRs and number of signal averages of the four acquired spectra were 0.7 s (TR_{short}), 1.7 s (TR_{center}), 1.7 s ($TR_{mirrored}$) and 2.7 s (TR_{long}) with 1024, 768, 768 and 548, respectively.

In order to determine k_f the FRiST method acquires 4 spectra. The partially saturated magnetization of P_i ($M'_{z,Pi}$) is measured at three different TRs: TR_{short}, TR_{center} and TR_{long} during continuous saturation of γ-ATP in order to calculate $T'_{1,Pi}$. As a fourth spectrum, $M_{0,Pi}$ is measured at TR_{center} in the presence of control irradiation applied at the mirrored frequency (further called mirrored spectrum). This compensates for possible spill over, originating from partially saturating the P_i resonance while aiming for the saturation of γ-ATP in the TR_{short}, TR_{center} and TR_{long} setup. From the difference in P_i amplitude between the spectrum with γ-ATP saturation and the spectrum with saturation at the mirrored frequency, the P_i exchange can be assessed.

The chemical shift between γ-ATP and P_i amounts ~7.7 ppm, resulting in a chemical shift displacement (CSD) of ~6.7 mm between these two metabolites. No respiratory triggering was used for the ST experiment. However, a sandbag was placed on the coil to restrict both coil and volunteer movement during the scans. 2D-localization was achieved by carefully placing a 40 × 90 × open mm^3 (AP,RL,FH) voxel of interest parallel to the coil in the transverse plane within the liver tissue, avoiding immediate proximity to the abdominal muscle (Supplemental Figure S1).

Supplemental material S4:

Data processing and quantification

Matlab script for processing of ST spectra

The script processes any number of spectra simultaneously and sorts them by their TR before fitting. All spectra were zero filled to 8192 points, apodized by a Gaussian filter of 18 Hz, frequency drift corrected, averaged and zero order phase optimized in the time domain by phasing spectra with the angle of the first complex data point. Hereby all spectra, independent of TR, were corrected with the overall mean phase angle. Subsequently, in total 13 resonances were fitted corresponding to all expected resonances in the obtained ^{31}P-MRS spectra. Briefly the following resonances were accounted for: α-, β-, γ-adenosine triphosphate (ATP), Glycero-phosphocholine (GPC), Glycero-phosphoethanolamine (GPE), inorganic phosphate (P_i), 2× nicotinamide adenine dinucleotide (NAD/NADH), Phosphocholine (PC), phosphocreatine (PCr), phosphoenolpyruvate/phosphatidylcholine (PEP/PtdC), Phosphoethanolamine (PE) and uridine diphosphate glucose (UDPG). PCr was included into the analysis to additionally account for potential contamination from abdominal muscles. All TR identical spectra were fitted with the same fitting model, incorporating T_2 estimates of resonances, J-coupling, and relative chemical shifts. A mix of Gaussian and Lorentzian line shapes was assumed and iteratively adjusted to match spectra. Furthermore, the fitting model takes the delay between excitation and acquisition of the FID into account and thereby fits the first order phase, making correction during post processing unnecessary.

Absolute quantification of P_i

^{31}P-spectra for absolute P_i quantification were processed using Java magnetic resonance user interface (jMRUI) software [8, 9] together with the advanced method for accurate, robust, and efficient spectral fitting (AMARES) [10, 11] algorithm and a custom created prior knowledge file was used for fitting as previously described [12].

Hepatic lipid content (HLC)

HL content was quantified as the ratio of intensities of the methylene (CH_2) peak in triglycerides at 1.3 ppm of liver ^{1}H-spectra to the combined signal intensities of the water and methylene peaks $HLC\ (\%) = \frac{S_{lipid}}{S_{lipid}+S_{water}}$. The difference in transverse relaxation times of water and lipid peaks was corrected based on a previous publication [13].

Supplemental material S5:

Theoretical background: Calculation of k_f

In order to calculate the pseudo-first order equilibrium forward rate constant (k_f) in human liver, a two-site model of chemical exchange between P_i and γ-ATP, as shown in equation 1, is typically assumed. Hepatic ATP is continuously synthesized from adenosine diphosphate (ADP) and P_i primarily in the mitochondria by oxidative phosphorylation, catalyzed by the enzyme ATP synthase:

$$ADP + P_i \underset{k_r}{\overset{k_f}{\rightleftharpoons}} ATP + H_2O \quad (1)$$

k_f and k_r represent the pseudo-first order equilibrium forward and reverse exchange rate constants, respectively.

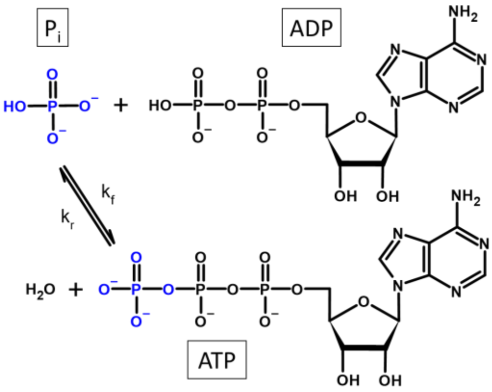

Schematic representation of adenosine triphosphate (ATP) synthesis. Adeonsine diphosphate (ADP) and inorganic phosphate (P_i) bind to ATP synthase, where they are joined to form ATP, releasing energy for cellular processes. k_f forward rate constant, k_r reverse rate constant.

During the ST experiment, the γ-ATP resonance is constantly saturated by frequency-selective irradiation, reducing the magnetization of γ-ATP to zero. Due to the chemical exchange described above, the equilibrium longitudinal magnetization of P_i is also reduced from its initial condition $M_{0,Pi}$ to $M'_{0,Pi}$ in presence of saturation. In addition, the spin-lattice relaxation time (T_1) of P_i also shifts from $T_{1,Pi}$ to an apparent $T'_{1,Pi}$ caused by chemical exchange of P_i with the saturated γ-ATP pool. From this, the longitudinal magnetization of P_i at an arbitrary time t during active saturation of γ-ATP, $M'_{Pi}(t)$, can be calculated by:

$$M'_{Pi}(t) = M'_{0,Pi} + \left(M'_{Pi}(t=0) - M'_{0,Pi}\right)e^{-\frac{t}{T'_{1,Pi}}} \quad (2)$$

$$\text{with } \frac{1}{T'_{1,Pi}} = \frac{1}{T_{1,Pi}^{intrinsic}} + k_f \text{ and } M'_{0,Pi} = \frac{M_{0,Pi}}{1+T_{1,Pi}^{intrinsic}\,k_f} \quad (3)$$

In equation 3, $T_{1,Pi}^{intrinsic}$ denotes the intrinsic longitudinal relaxation time of P_i in case no exchange is present. From these formulas, one can deduce the reaction rate:

$$k_f = \frac{1}{T'_{1,Pi}}\left(1 - \frac{M'_{0,Pi}}{M_{0,Pi}}\right) \tag{4}$$

In summary, three variables are required to assess k_f: The apparent spin-lattice relaxation time of P_i ($T'_{1,Pi}$), the equilibrium ($M_{0,Pi}$) and apparent longitudinal magnetization of P_i ($M'_{0,Pi}$).

Supplemental Fig. S1:

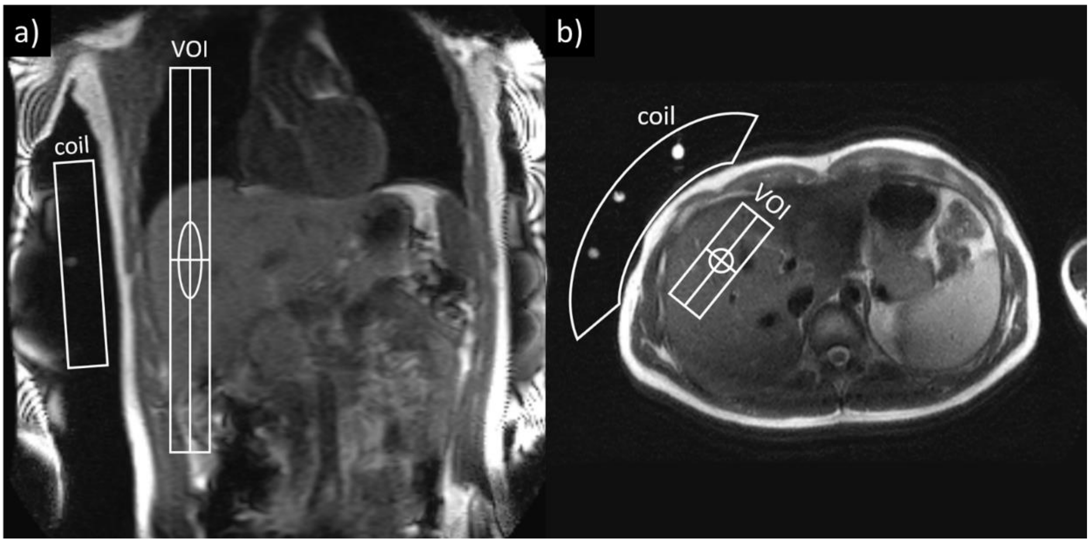

Supplemental Fig. S1: Planning of the FRiST protocol. a) Coronal and b) transverse MRI slices of the liver of a 30 years old control volunteer showing coil placement and position of the 2D-voxel of interest (VOI) (open in feet-head direction). White dots within the coil housing show position of reference spheres. *FRiST* Four Repetition time Saturation Transfer.

Supplemental Fig. S2:

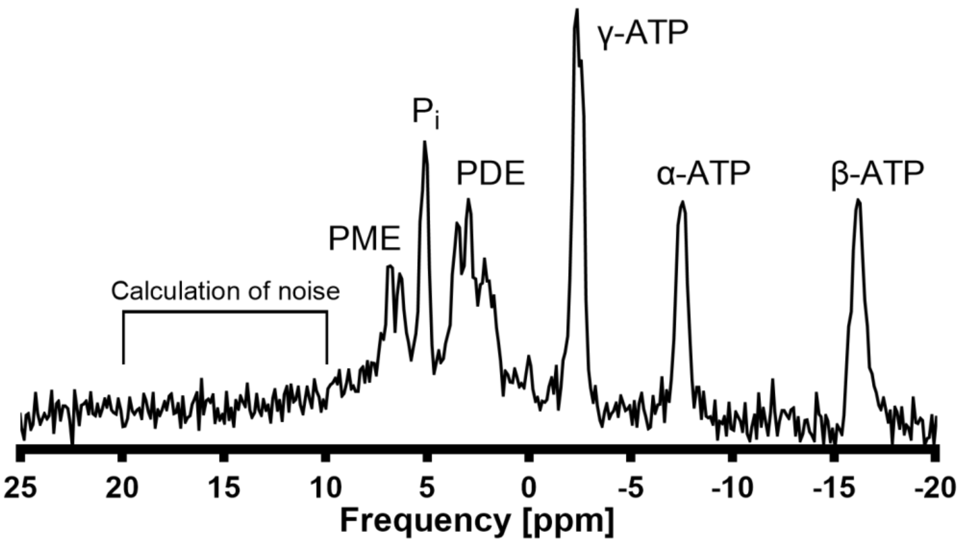

Supplemental Fig. S2: Representative raw hepatic phosphorus magnetic resonance spectroscopy spectrum. No postprocessing was performed except zero order phasing. The spectral region between 10 ppm and 20 ppm served to determine the noise for calculation of signal-to-noise for spectral quality assessment. *ATP* adenosine triphosphate, *PDE* phosphodiesters, P_i inorganic phosphate, *PME* phosphomonoesters.

Supplemental Fig. S3:

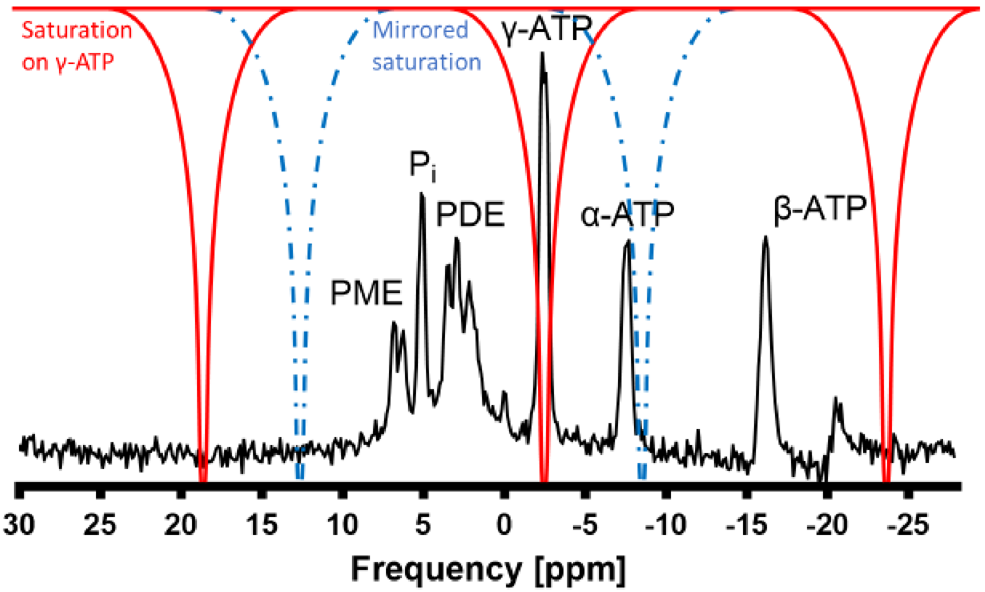

Supplemental Fig. S3: Position of the delays alternating with nutations for tailored excitation (DANTE) saturation bands with m = 3 saturation bands which were δ = 12 Hz (M3D12) apart on an entire hepatic phosphorus magnetic resonance spectroscopy spectrum. In the saturation experiment, the saturation pulse was centered on the γ-ATP resonance at ~-2.48 ppm (saturation bands displayed in red). Two aliased saturation bands (~-23.6 ppm and ~18.7 ppm) occur every ~21.2 ppm (1/τ = 1100 Hz) from the center of saturation frequency due to a DANTE subpulse duration of τ = 0.91 ms. In the mirrored experiment, the mirrored saturation frequency was set to ~12.7 ppm resulting in aliased saturation bands at ~33.82 ppm (not shown) and ~-8.5 ppm (blue dashed lines). Note that in the mirrored experiment α-ATP is partly affected by the aliased sideband resulting in a decreased signal amplitude. For better representation, the spectrum is apodized with a 5 Hz Gaussian filter.
ATP adenosine triphosphate, *PDE* phosphodiesters, P_i inorganic phosphate, *PME* phosphomonoesters.

Supplemental Table S1:

Supplemental Table S1: MRSinMRS checklist [14].

German Diabetes Center Düsseldorf	^{31}P MRS ST	^{31}P MRS	^{1}H MRS
**1. Hardware**			
a. Field strength [T]	3T		
b. Manufacturer	Philips, Best, the Netherlands		
c. Model (software version if available)	Achieva 3T dStream, R5.6/R.5.7		
d. RF coils: nuclei (transmit/ receive), number of channels, type, body part	^{31}P curved quadrature surface coil, total loop size 220 × 160 mm^2, transmit & receive, Rapid Biomedical		Body coil, Philips, Best, the Netherlands
e. Additional hardware	-		
**2. Acquisition**			
a. Pulse sequence	Basic pulse sequence: Image Selected In Vivo Spectroscopy (ISIS), vendor supplied<br>Research patch added implementation of frequency-selective DANTE saturation pulse trains		STimulated Echo Acquisition Mode (STEAM), vendor supplied
b. Volume of Interest (VOI) locations	Lateral side of liver		
c. Nominal VOI size	40 × 90 × 300 mm^3 (AP,RL,FH)	60 × 60 × 60 mm^3	25 × 25 × 25 mm^3
d. Repetition Time (TR), Echo Time (TE) [ms, s]	TR 700, 1700, 2700 ms, TE 0.096 ms	TR 6000 ms,<br>TE 0.096 ms	TR 4000 ms,<br>TE 10 ms
e. Total number of Excitations or acquisitions per spectrum	TR 700 ms:<br>1024 NSA<br>TR 1700 ms:<br>768 NSA<br>TR 2700 ms:<br>548 NSA	128 NSA	16 NSA for both non-water suppressed and water suppressed spectra
f. Additional sequence parameters	Bandwidth 3000 Hz<br>Datapoints 512	Bandwidth 3000 Hz<br>Datapoints 2048	Bandwidth 2000 Hz<br>Datapoints 1024

(spectral width in Hz, number of spectral points, frequency offsets)	Excitation pulse center was set exactly in between γ-ATP and P_i<br>Chemical shift displacement: γ-ATP ↔ Pi = ~7.7 ppm → ~6.7 mm	Broadband decoupling (WALTZ-4, B_1 ampl. 7 μT)<br>Continuous wave NOE with mixing time 3500 ms, B_1 ampl. 0.5 μT<br>Excitation pulse center was set to -1.0 ppm (between γ-ATP and P_i)<br>Chemical shift displacement: γ-ATP ↔ Pi = ~7.7 ppm → ~6.7 mm	TM 16 ms<br>Excitation pulse center was set to water frequency
g. Water Suppression Method	-		CHEmical Shift Selective saturation (CHESS)
h. Shimming Method, reference peak, and thresholds for “acceptance of shim” chosen	3D-FFE, 1 BH<br>FOV 350 × 292 × 160 mm^3, acq. voxel size 5 × 5 × 10 mm^3, rec. voxel size 2.43 × 2.43 × 10 mm^3, 16 transverse slices	Vendor second order pencil beam VOI (shim size 60 × 60 × 60 mm^3)<br>System reported FWHM between 20 and 45 Hz	Vendor second order pencil beam VOI (shim size 50 × 50 × 50 mm^3)
i. Triggering or motion correction method	-		
**3. Data analysis methods and outputs**			
a. Analysis software	Matlab	jMRUI	Matlab
b. Processing steps deviating from quoted reference or product	Gaussian apodization 18 Hz, zero filling to 8192 points, frequency drift corrected, zero order phase optimization	Gaussian apodization 15 Hz, frequency adjustment to γ-ATP = -2.48 ppm, zero order phasing	Gaussian apodization 15 Hz, zero filling to 4096 points, frequency adjustment to water = 4.7ppm or methylene = 1.3ppm

c. Output measure<br>(e.g. absolute concentration, institutional units, ratio) Processing steps deviating from quoted reference or product	P_i amplitudes [a.u.]	Absolute units (millimolar [mM]) Correction for T_1, individual metabolite frequency offset, coil loading and distance in all three dimensions	Hepatic lipid content (HLC) [%]
d. Quantification references and assumptions, fitting model assumptions	13 resonances were fitted with a mix of Gaussian and Lorentzian line shapes incorporating T_2 estimates of resonances, J-coupling, relative chemical shifts	AMARES in jMRUI, custom created prior knowledge (Gaussian line shapes, estimated amplitudes, fixed relative phases, individual line width and chemical frequencies kept in soft constraints	22 resonances were fitted in the water spectrum and 23 in the water-suppressed spectrum with a mix of Gaussian and Lorentzian line shapes incorporating T_2 estimates of resonances, J-coupling, relative chemical shifts
4. Data Quality			
a. Reported variables<br>(SNR, Linewidth (with reference peaks))	Good quality data: SNR (γ-ATP and P_i)<br>Potential signal contamination from abdominal muscle: PCr/γ-ATP peak ratio<br>Only for ST: Degree of γ-ATP saturation		-
b. Data exclusion criteria	SNR of γ-ATP and P_i:<br>SNR: γ-ATP < 4<br>SNR: P_i < 2.5<br>Potential signal contamination from abdominal muscle: PCr/γ-ATP > 0.5	SNR of γ-ATP and P_i:<br>SNR: γ-ATP < 4<br>SNR: P_i < 2.5<br>Potential signal contamination from abdominal muscle: PCr/γ-ATP > 0.5<br>Spectra were excluded for distances between coil and center of ISIS voxel > 13.5 cm	Malfunction of water-suppression
c. Quality measures of postprocessing Model fitting (e.g. CRLB,	-	-	-

goodness of fit, SD of residual)			
d. Sample Spectrum	Figure 4 & Figure 5	See 10.1002/nbm.5120	See 10.1038/s41467-020-15684-0

Supplemental Table S2:

Supplemental Table S2: 3TR versus 5TR. Results of apparent spin-lattice relaxation time ($T'_{1,Pi}$) fitting of a saturation experiment to find the best suited three repetition time (TR) set. Two control volunteers underwent a saturation recovery experiment consisting of five TRs. The experimentally determined $T'_{1,Pi}$ together with the calculated coefficient of determination (R^2) were compared to find the most accurate set of three TRs.

TR combination [ms]	Control volunteer #1		Control volunteer #2	
	$T'_{1,Pi}$ [ms]	R^2	$T'_{1,Pi}$ [ms]	R^2
700, 1200, 1700, 2200 & 2700	699	0.9962	432	0.9962
700, 1200 & 1700	598	0.9996	492	1
700, 1200 & 2200	743	0.9975	402	0.9978
700, 1200 & 2700	666	0.9989	455	0.9996
700, 1700 & 2200	693	0.9969	430	0.9964
700, 1700 & 2700	645	0.9993	465	0.9995
700, 2200 & 2700	665	0.9986	433	0.9982

Supplemental Table S3:

Supplemental Table S3: Calculated phosphorus-MR spectroscopy parameters for the subgroups of control volunteers and type 1 diabetes using ANOVA adjusted for age, sex and BMI.

	Control volunteers	Type 1 diabetes	*p*-value
Number	9	8	
$T'_{1,Pi}$ [ms]	488 [419; 557]	513 [437; 588]	0.640
ΔM [%]	16.0 [13.2; 18.8]	8.3 [5.3; 11.3]	0.003*
k_f [s^{-1}]	0.33 [0.26; 0.41]	0.18 [0.09; 0.26]	0.017*
F_{ATP} [mM/min]	35.4 [27.3; 43.4]	18.3 [9.6; 27.1]	0.014*

All values are reported as numbers of participants or mean [95% CI]. *$p < 0.05$ indicates statistical significance

ΔM Percentage difference in P_i amplitude between saturating and control irradiation, F_{ATP} Forward adenosine triphosphate synthesis rate, k_f Forward rate constant, P_i Inorganic phosphate, $T'_{1,Pi}$ Apparent spin-lattice relaxation time of P_i.

References

1. Szendroedi J, Saxena A, Weber KS et al (2016) Cohort profile: the German Diabetes Study (GDS). Cardiovasc Diabetol 15:59. https://doi.org/10.1186/s12933-016-0374-9
2. Schär M, El-Sharkawy A-MM, Weiss RG et al (2010) Triple repetition time saturation transfer (TRiST) ^{31}P spectroscopy for measuring human creatine kinase reaction kinetics. Magn Reson Med 63:1493–1501. https://doi.org/10.1002/mrm.22347
3. Eberhardt KW, Schär M, Barmet C et al (2006) Linear response equilibrium. J Magn Reson 178:142–154. https://doi.org/10.1016/j.jmr.2005.09.005
4. Ordidge R, Connelly A, Lohman J (1986) Image-selected in vivo spectroscopy (ISIS). A new technique for spatially selective NMR spectroscopy. J Magn Reson 66:283–294. https://doi.org/10.1016/0022-2364(86)90031-4
5. Bottomley PA, Ouwerkerk R, Lee RF et al (2002) Four-angle saturation transfer (FAST) method for measuring creatine kinase reaction rates in vivo. Magn Reson Med 47:850–863. https://doi.org/10.1002/mrm.10130
6. Schmid AI, Chmelík M, Szendroedi J et al (2008) Quantitative ATP synthesis in human liver measured by localized ^{31}P spectroscopy using the magnetization transfer experiment. NMR Biomed 21:437–443. https://doi.org/10.1002/nbm.1207
7. Buehler T, Kreis R, Boesch C (2015) Comparison of ^{31}P saturation and inversion magnetization transfer in human liver and skeletal muscle using a clinical MR system and surface coils. NMR Biomed 28:188–199. https://doi.org/10.1002/nbm.3242
8. Naressi A, Couturier C, Castang I et al (2001) Java-based graphical user interface for MRUI, a software package for quantitation of in vivo/medical magnetic resonance spectroscopy signals. Comput Biol Med 31:269–286. https://doi.org/10.1007/BF02668096
9. Stefan D, Di Cesare F, Andrasescu A et al (2009) Quantitation of magnetic resonance spectroscopy signals: the jMRUI software package. Meas Sci Technol 20:104035. https://doi.org/10.1088/0957-0233/20/10/104035
10. Mierisová Š, van den Boogaart A, Tkáč I et al (1998) New approach for quantitation of short echo time in vivo 1H MR spectra of brain using AMARES. NMR Biomed 11:32–39. https://doi.org/10.1002/(sici)1099-1492(199802)11:1<32:aid-nbm501>3.0.co;2-
11. Vanhamme L, van den Boogaart A, van Huffel S (1997) Improved method for accurate and efficient quantification of MRS data with use of prior knowledge. J Magn Reson 129:35–43. https://doi.org/10.1006/jmre.1997.1244
12. Jonuscheit M, Wierichs S, Rothe M et al (2024) Reproducibility of absolute quantification of adenosine triphosphate and inorganic phosphate in the liver with localized ^{31}P-magnetic resonance spectroscopy at 3-T using different coils. NMR Biomed 37:e5120. https://doi.org/10.1002/nbm.5120
13. Hamilton G, Yokoo T, Bydder M et al (2011) In vivo characterization of the liver fat ^{1}H MR spectrum. NMR Biomed 24:784–790. https://doi.org/10.1002/nbm.1622
14. Lin A, Andronesi O, Bogner W et al (2021) Minimum reporting standards for in vivo magnetic resonance spectroscopy (MRSinMRS): experts' consensus recommendations. NMR Biomed 34:e4484. https://doi.org/10.1002/nbm.4484
